# Causal association between kynurenine and depression investigated using two-sample mendelian randomization

**DOI:** 10.1038/s41598-024-52442-4

**Published:** 2024-01-20

**Authors:** Luxin Zong, Maohong Ge, Jiamiao Wang, Dan Kuang, Hongkai Wei, Zhongbao Wang, Zerui Hu, Chaoben Zhao, Qingmin Jin, Minghui Chen, Chenghui Wang

**Affiliations:** 1 Mental Health Center of Weifang City, No. 8899, Wei’an Road, High-tech Zone, Weifang, 26100 Shandong Province People’s Republic of China; 2 School of Clinical Medicine, Weifang Medical University, Weifang, People’s Republic of China; 3 Shaoxing Seventh People’s Hospital, Shaoxing, People’s Republic of China; 4 Department of Psychiatry, Shandong Daizhuang Hospital, Jining, People’s Republic of China

**Keywords:** Clinical genetics, Genetics, Neuroscience, Medical research, Neurology, Risk factors

## Abstract

As research progresses, the intricate metabolic connections between depression and tryptophan, as well as kynurenine (KYN), have become increasingly evident. In studies investigating the relationship between KYN and depression, the conclusions reached thus far have been inconsistent. Therefore, we propose employing a two-sample mendelian randomization (MR) approach to further elucidate the relationship between KYN and depression. We utilized extensive data from large-scale genome-wide association studies to identify single nucleotide polymorphisms that act as instrumental variables for kynurenine and depression in European ancestry populations, ensuring compliance with MR assumptions. We employed five MR algorithms, namely, weighted median, MR-Egger, inverse variance weighted (IVW), simple mode, and weighted mode, with IVW as the primary analysis method. Sensitivity tests were conducted using Cochran’s Q test, MR-Egger intercept test, MR Pleiotropy Residual Sum and Outlier, and Leave-one-out analysis.The IVW analysis revealed that each standard deviation increase in kynurenine corresponded to a 1.4-fold increase in the risk of depression (OR = 1.351, 95% CI 1.110–1.645, *P* = 0.003). The direction of the effect size (positive or negative) was consistent with the findings from the other four algorithms. Sensitivity tests indicated no heterogeneity or horizontal pleiotropy among the instrumental variables. Elevated levels of kynurenine have a causal relationship with an increased risk of developing depression.

## Introduction

Depression is a prevalent mental disorder characterized by persistent low mood, reduced interest, and diminished pleasure, which can disrupt eating and sleeping patterns and, in severe cases, lead to suicide. The global incidence of depression has increased by almost 50% over the past 30 years, affecting more than 264 million people worldwide^1^. Of particular concern is the recurrent nature of depression^2^. Tryptophan metabolism, particularly involving serotonin (5-HT) and kynurenine (KYN), plays a crucial role in the development of depression. It is responsible for synthesizing essential neuroactive compounds, 5-HT, and KYN^3–6^. Antidepressants that target the reuptake of 5-HT in the synaptic cleft have proven effective and are among the most widely used medications worldwide for treating depression, highlighting the significance of 5-HT in depressive disorders^4^. In contrast, research on KYN is relatively limited. As investigations progress, the metabolic connections between tryptophan, 5-HT, and KYN are becoming increasingly intricate in the context of depression. Despite the extensive research on the relationship between KYN and depression, conclusions are inconsistent. Some studies indicate lower levels of KYN in individuals with depression compared to control groups, while others report no association between depression and KYN levels^7,8^. Additionally, certain studies suggest elevated KYN levels in individuals with depression^9^.

Mendelian randomization (MR) is a method that utilizes data from genome-wide association studies (GWAS) and leverages genetic variants, specifically single nucleotide polymorphisms (SNPs), strongly associated with an exposure (such as KYN) as instrumental variables (IVs) to determine the causal relationship between the exposure and the outcome of interest (such as depression). By relying on the random distribution of genetic variants, MR is less prone to confounding factors and reverse causality, reducing bias and resembling randomized controlled trials. Hence, the objective of this study is to elucidate the causal relationship between KYN and depression through a stringent two-sample MR analysis.

## Method

### Data source

The GWAS data for depression and KYN: Both are derived from the European population’s GWAS from IEU Open GWAS (https://gwas.mrcieu.ac.uk/), with GWAS IDs for depression and KYN being ieu-b-102 and met-a-375, respectively. The GWAS data for depression originate from a study involving 170,756 depression patients and 329,443 controls^10^. The GWAS data for KYN originate from a human blood metabolite study with a sample size of 7816^11^. Refer to Table 1 for specific details.

**Table 1:** Sample sizes, age groups and the proportion of males and females of the study cohorts.

Items	Study	Sample size (prop. male, female)		Cohort	Age groups (mean)
		Cases	Controls		
Depression^†^	Howard et al.^10^	170,756 (–)	329,443 (–)	UK biobank	–
				PGC	
KYN	Shin et al.^11^	7,816 (0.17, 0.83)		KORA	32–77 (61)
				TwinsUK	17–85 (53)

^†^The distribution of age and gender not reported for the depression cohorts; PGC, Psychiatric Genomics Consortium; KORA, Cooperative Health Research in the Region of Augsburg.

This study reanalyzes public data and does not require ethical approval.

### Method

#### Selection of instrumental variables

To perform two-sample MR analysis and investigate the causal relationship between KYN and depression, IVs need to meet certain assumptions: (1) The SNPs were significantly correlated with the exposure; (2) SNPs can only influence the outcome through exposure. The following steps were taken to select suitable IVs:

Single nucleotide SNPs associated with exposure: SNPs were selected from the GWAS data for KYN based on their association with KYN at a significance threshold of *P* < 5 × 10^–6^ (If SNPs are filtered based on a threshold of *P* < 5 × 10^–8^, after the exclusion of weak instruments and the removal of confounding steps, we only retained 2 SNPs, making it unfeasible to conduct MR analysis). SNPs exhibiting genomic linkage imbalance (distance window < 10,000 kb, linkage disequilibrium coefficient r^2^ < 0.001) were removed. The remaining SNPs were assessed for their F-statistic, and SNPs with F < 10, indicating weak instrumental variables, were excluded. Only SNPs with F > 10, demonstrating a significant association with the exposure, were retained^12^. The F-statistic was calculated using the formula F = (N−K−1) * R^2^/(1−R^2^), R^2^ = β^2^ (1—EAF) * 2EAF / SD^2^, where R^2^ represents the proportion of variation explained by each SNP, K is the number of SNPs, N is the total sample size, EAF is the allele frequency of the mutation, β is the beta coefficient related to the exposure factor, and SD is the variance^13^.

SNPs independent of outcome: SNPs associated with the outcome (depression) at a significance threshold of *P* < 5 × 10^–8^ were removed from the IVs. Palindromic variants with identical base pairs were also excluded to avoid potential double-counting of their variation. The remaining SNPs were verified against Phenoscanner (http://www.phenoscanner.medschl.cam.ac.uk/) to identify and exclude those influenced by confounding factors. The remaining SNPs meeting the MR assumptions were used as instrumental variables.

### MR analysis

MR analysis was performed using five methods: Weighted median (WM), MR-Egger, inverse variance weighted (IVW), simple mode, and weighted mode. The IVW method served as the primary analysis. A significant result from the IVW analysis (*P* < 0.05) along with consistent effect directions (positive or negative) across the other four methods supports the inference of a causal relationship. This means that the results of these five algorithms are consistent, supporting the remarkable results of IVW. The IVW algorithm is a commonly used two-sample MR analysis method, which can make full use of the sample information of each study, thereby improving statistical power. When using other algorithms, some preprocessing of the data may be required, such as removing outliers, performing data transformation, etc., and these preprocessing may affect the results. The IVW algorithm requires less data and does not require special preprocessing, so the results may be more accurate. In addition, if the sample size is small or the sample distribution is uneven, the results of other algorithms may be more affected, and the IVW algorithm can maintain high statistical power in this case. When all SNPs adhere to the assumptions of MR, only under such conditions can the inverse variance weighted (IVW) method yield accurate estimates of causal relationships. The WM method leverages a majority of effective instrumental variables (IVs) for causal inference. MR-Egger, on the other hand, allows the inclusion of IVs that exhibit pleiotropy. The intercept is used to gauge horizontal pleiotropy, while the slope serves to ascertain causal relationships. A *P*-value less than 0.05 signifies that the results of the MR analysis carry significant statistical importance. The “TwoSampleMR” package in R 4.1.2 was utilized for all data processing.

### Sensitivity analysis

Sensitivity tests were carried out to gauge the robustness of the findings. Cochran’s Q test was implemented to assess the heterogeneity among SNPs, with a *P*-value of less than 0.05 indicating significant heterogeneity. Techniques such as the MR-Egger intercept test and MR Pleiotropy Residual Sum and Outlier (MR-PRESSO) were deployed to investigate horizontal pleiotropy. If the intercept in the MR-Egger intercept test is statistically significant (*P* < 0.05), it implies a significant presence of pleiotropy. If the *P*-value of the MR-PRESSO result falls below 0.05, it suggests that the MR analysis presents considerable horizontal pleiotropy, which means that the IVs can directly influence the outcome without acting through the exposure factor. A leave-one-out analysis was conducted, which involved the sequential exclusion of each SNP to examine the impact on the combined effect of the remaining SNPs. Any notable alterations in the results due to the exclusion of a particular SNP suggests that the SNP has a substantial influence on the result, which infringes on the assumption of MR. The final results were presented using scatter plots, forest plots, and funnel plots.

### Institutional review board statement

This study was based on a published database and did not require ethical approval.

## Results

The MR analysis examined the causal relationship between KYN as the exposure and depression as the outcome. After applying the selection criteria, a total of 17 SNPs were identified as IVs that met the MR assumptions. Eight SNPs with F < 10 were excluded from the analysis, namely, rs3809198 (F = 0.83), rs16924894 (F = 1.46), rs16974854 (F = 1.74), rs11593042 (F = 3.50), rs511797 (F = 4.70), rs3789978 (F = 6.28), rs6575634 (F = 9.63), and rs2651516 (F = 9.97). No SNPs significantly associated with depression (*P* < 5 × 10^–8^) were found among the KYN SNPs extracted from the depression GWAS data. Palindromic variants were not detected. However, the SNP rs3184504 was identified as confounded by body mass index, which has been causally linked to depression in previous MR studies. Hence, this SNP was removed from the analysis^14^. Detailed results are presented in Table 2.

**Table 2:** Information on the SNPs for the final screened KYN (n = 17).

SNP	Gene	Effect allele	Other allele	β	SE	*P*	*P*^a^	F
rs10085935	IDO2	T	C	− 0.010	0.002	3.33E−09	0.365	17.30
rs10857319	FNIP2	T	C	− 0.009	0.002	9.45E−07	0.208	11.63
rs11646849	CDH13	A	G	− 0.009	0.002	6.40E−07	0.865	12.81
rs12082398	–	C	T	− 0.012	0.002	9.33E−07	0.545	11.55
rs12937634	ZNF652	C	T	0.008	0.002	2.28E−06	0.028	11.57
rs1426134	RP11-669M16.1	G	C	0.008	0.002	3.84E−06	0.664	10.61
rs1496635	LINC01791	C	A	0.009	0.002	2.12E−07	0.126	11.50
rs21327	NBPF13P	G	A	− 0.009	0.002	1.25E−07	0.766	11.39
rs2320536	MAML3	T	C	0.008	0.002	3.32E−06	0.432	11.02
rs2375475	RAVER2	T	C	− 0.008	0.002	1.41E−06	0.265	10.70
rs2491294	RP11-60A14.1	G	A	0.01	0.002	5.73E−07	0.132	10.71
rs4820242	CACN2	A	G	− 0.009	0.002	9.03E−07	0.578	11.79
rs6770323	SLC2A2	T	C	− 0.008	0.002	3.33E−06	0.890	10.87
rs6815057	MANBA	A	G	0.009	0.002	1.12E−06	0.169	12.21
rs7548008	CLDN19	G	A	− 0.016	0.004	3.56E−06	0.856	10.46
rs8051149	SLC7A5	A	G	0.026	0.003	9.07E−26	0.035	35.82
rs9857268	HSPE1P19	A	G	0.008	0.002	3.07E−06	0.358	10.73

SNP, single nucleotide polymorphism; Gene, Gene information is derived from Phenoscanner; β, effector value; SE, standard error; *P*^a^, the *P* value of depression SNPs.

The IVW method, serving as the primary analysis, indicated that each standard deviation increase in KYN was associated with an approximately 1.4-fold increase in the risk of depression (OR = 1.351, 95% CI 1.110–1.645, *P* = 0.003). The effect directions from the other four methods were consistent with IVW. Further details can be found in Table 3.

**Table 3:** Results of the five MR algorithms.

MR algorithms	Effect value	*P*	OR (95% CI)
MR-Egger	0.372	0.196	1.451 (0.846–2.487)
WM	0.389	0.006	1.476 (1.116–1.951)
IVW	0.301	0.003	1.351 (1.110–1.645)
Simple mode	0.335	0.186	1.398 (0.869–2.247)
Weighted mode	0.335	0.085	1.398 (0.977–1.998)

WM, Weighted median; IVW, inverse variance weighted.

**Figure 1:**
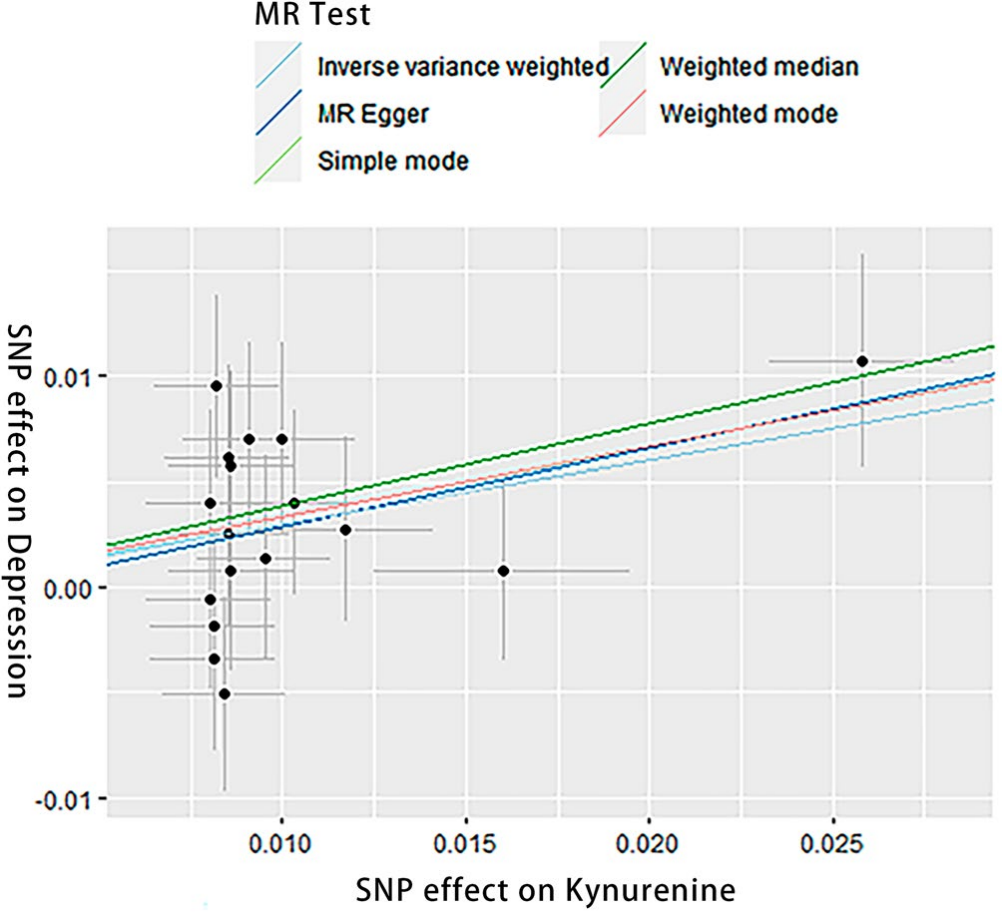
Scatter plot (n = 17).

**Figure 2:**
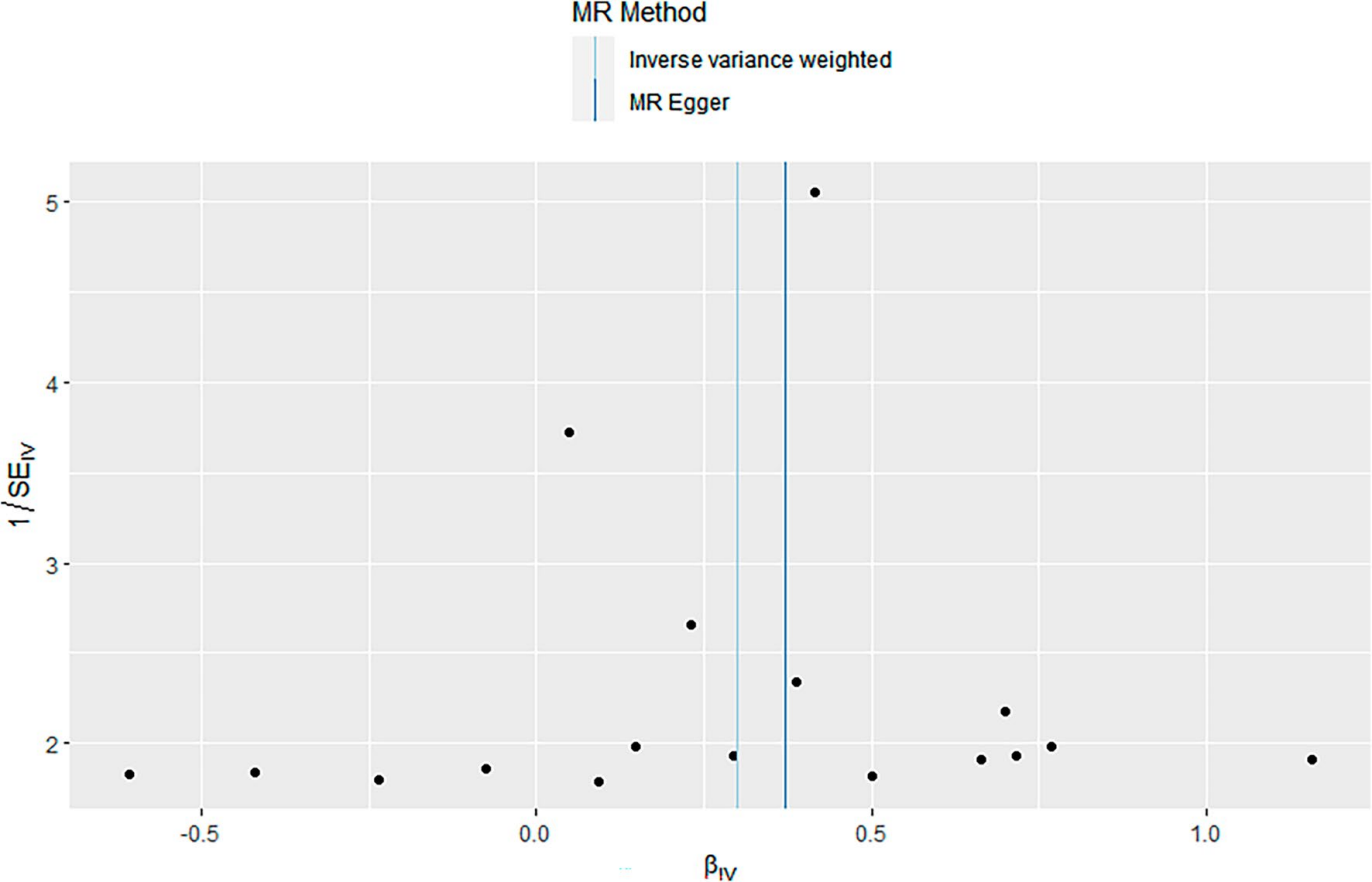
Funnel plot (n = 17).

**Figure 3:**
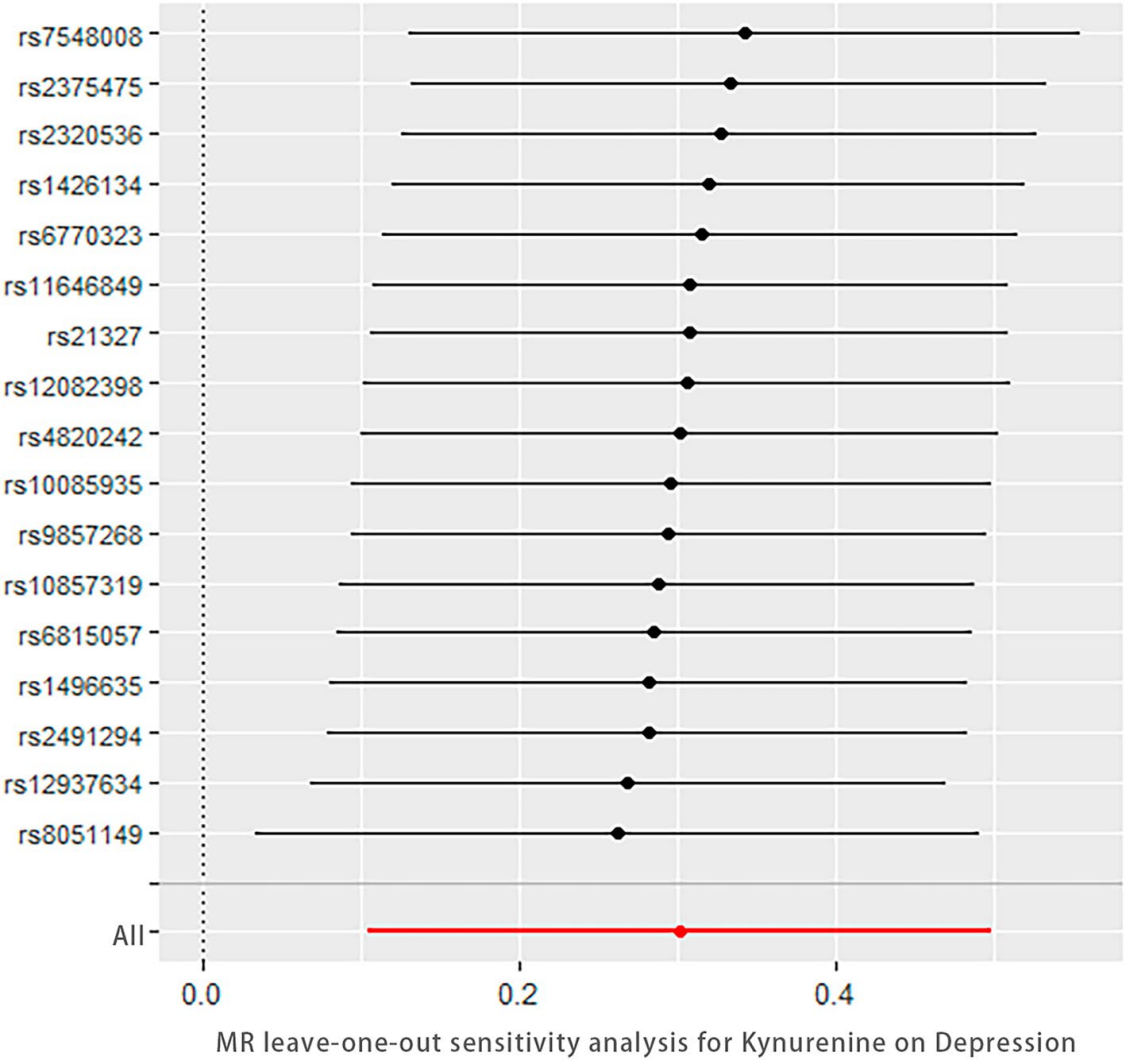
Forest plot of the leave-one-out analysis (n = 17).

Sensitivity analysis demonstrated no heterogeneity among the SNPs, as indicated by Cochran’s Q test (IVW: Q = 12.982, *P* = 0.674; MR-Egger: Q = 12.905, *P* = 0.610). The MR-Egger intercept test showed no evidence of horizontal pleiotropy (Intercept = − 0.001, *P* = 0.785). MR-PRESSO analysis did not identify any outliers (*P* = 0.719). The scatter plot illustrated the stability of the SNPs strongly associated with both KYN and depression (Fig. 1). The funnel plot displayed a symmetrical distribution of the SNPs, indicating no pleiotropy in the MR analysis (Fig. 2). Leave-one-out analysis demonstrated that the exclusion of individual SNPs did not significantly impact the overall results (Fig. 3).

## Discussion

Emerging evidence points to the involvement of KYN, a metabolite of tryptophan, in the development of depression, with disruptions in the kynurenine pathway playing a significant role in the etiology of depression^15,16^. However, the relationship between KYN and depression remains unclear. This study endeavored to explore the causal relationship between KYN and depression using a two-sample MR approach. The research results provide robust evidence, indicating that an elevation in KYN levels significantly increases the risk of developing depression.

The chronic inflammation hypothesis of depression has garnered substantial literature support, positing depression as a neuroimmune disorder where the activation of the immune system plays a pivotal role in the onset and progression of depression^17–19^. Neuroinflammation is implicated in the regulation of the tryptophan-kynurenine (TRP-KYN) pathway, particularly modulated by various pro-inflammatory cytokines such as TNF-α, IL-6, INF-γ^20,21^. Previous observational studies have reported associations between abnormalities in tryptophan-kynurenine metabolism and depressive disorders, particularly in scenarios involving inflammation. These studies align with our findings, which demonstrate an increase in KYN levels in individuals with depression^9,11,22–25^. Some scholars have proposed a novel subtype of depression termed “immune-metabolic depression”, wherein alterations in inflammation, metabolism, and bioenergetic pathways are observed in the majority of individuals with depression^26^. Regrettably, given the current unavailability of GWAS data for immune-metabolic depression, we are unable to elucidate the causal relationship between KYN and immune-metabolic depression. If GWAS data for immune-metabolic depression and non-immune-metabolic depression become accessible in the future, a renewed MR analysis could be conducted to investigate the causal relationship between KYN and these two subtypes of depression. KYN may lean towards having a causal relationship with immune-metabolic depression. Future research might explore common genetic features between KYN and immune-metabolic depression in this direction.

Potential mechanisms underlying the link between KYN and depression may involve the induction of depressive-like behavior through increased expression of Nod-like receptor protein 2 (NLPR2) in astrocytes by KYN, mediated by proinflammatory cytokines^4^. Cytokines such as IL-1β, IL-6, and TNF-α can also inhibit the conversion of KYN to 5-HT by activating the kynurenine pathway, resulting in a decrease in 5-HT synthesis and the manifestation of depressive symptoms^27^.

Several limitations should be considered in interpreting the findings of this study. First, the data used in this analysis were derived from individuals of European ancestry, potentially limiting the generalizability of the results to other populations. Second, while various sensitivity analyses were conducted to assess the assumptions of the MR study, it is challenging to completely rule out the possibility of pleiotropy influencing the instrumental variables. Last, the sample size of the available GWAS datasets was limited, emphasizing the need for further research using larger and more diverse datasets.

## Conclusions

In this study, we utilized a two-sample MR approach to investigate the causal relationship between KYN and depression. The findings provide compelling genetic evidence supporting a causal link between elevated levels of KYN and an increased risk of depression. This provides genetic evidence for understanding the etiological mechanisms of depression and potential interventions. Specifically, it prompts further exploration into whether the dysregulation of the KYN metabolic pathway is a pathogenic mechanism for depression, whether KYN can serve as a potential therapeutic target, or whether it can be used as a diagnostic or screening marker for depression. However, these findings warrant further investigation and validation.

## Data Availability

Publicly available datasets were analyzed in this study. These data can be found in the the MRC IEU Open GWAS Database. The script repository for this study is located at: https://github.com/zlxwy163/MR-Code/tree/main
